# Factors associated with socioeconomic inequalities in the prevalence of chronic NCD risk factors in the DIMAMO surveillance site, South Africa

**DOI:** 10.1371/journal.pgph.0007483

**Published:** 2026-10-26

**Authors:** Khuliso Goodman Ravhuhali, Cairo Bruce Ntimana, Eric Maimela

**Affiliations:** 1 Department of Public Health, University of Limpopo, Polokwane, South Africa; 2 National Health Laboratory Service, Johannesburg, South Africa; 3 DIMAMO Population Health Research Centre, University of Limpopo, Polokwane, South Africa; 4 Department of Public Health, Faculty of Medicine and Health Sciences, Walter Sisulu University, Eastern Cape, South Africa; Johns Hopkins School of Medicine: The Johns Hopkins University School of Medicine, UNITED STATES OF AMERICA

## Abstract

Non-communicable diseases (NCDs) are a leading cause of morbidity and mortality globally, with a disproportionate burden in low- and middle-income countries and among socioeconomically disadvantaged populations. Understanding socioeconomic inequalities in NCD risk factors is important for developing targeted prevention strategies. This study assessed factors associated with socioeconomic inequalities in chronic NCD risk factors among adults in the DIMAMO Health and Demographic Surveillance System (HDSS) in rural South Africa. A cross-sectional survey was conducted among 398 adults aged ≥18 years attending primary healthcare facilities within the DIMAMO HDSS catchment area. SES was measured using an asset-based wealth index derived through principal component analysis and categorized into quintiles. Data on tobacco use, harmful alcohol use, physical inactivity, low fruit and vegetable intake, high salt intake, self-reported hypertension, and diabetes, were collected using adapted WHO STEPS and SAGE questionnaires. Crude and adjusted prevalence ratios (PRs and aPRs) were estimated using modified Poisson regression with robust standard errors. SES was not significantly associated with hypertension after adjustment. Participants in the richer (aPR = 1.58; 95% CI: 1.03–2.41) and richest (aPR = 1.78; 95% CI: 1.11–2.85) SES quintiles had a higher prevalence of harmful alcohol use than those in the poorest quintile. Physical inactivity was less prevalent among participants in the middle, richer, and richest quintiles. Tobacco use was less prevalent among females and participants with secondary or tertiary education. Self-reported diabetes tended to be more prevalent among richest quintile, although the association was not statistically significant. Age, sex, educational attainment, and employment status were associated with several NCD risk factors. Socioeconomic inequalities in NCD risk factors were evident in this rural population. Higher SES was associated with harmful alcohol use and a lower physical inactivity. These findings highlight complex socioeconomic patterning of NCD risk factors and support context-specific interventions and longitudinal research.

## 1. Introduction

Non-communicable diseases (NCDs) are the leading causes of morbidity globally and account for seven of the ten leading causes of death worldwide [1–3]. Recent global burden estimates identify metabolic, behavioural, environmental, and dietary risks as major contributors to NCD morbidity and mortality [3,4]. The burden is particularly important in low- and middle-income countries (LMICs), where rapid demographic, epidemiological, and nutrition transitions are occurring alongside persistent health-system constraints and socioeconomic inequalities [1,5,6].

Socioeconomic status (SES) is increasingly recognised as an important determinant of the distribution of NCDs and their risk factors, but the direction of this association differs by outcome, country income level, and stage of transition [5–7]. Recent LMIC evidence shows that lifestyle-related risk factors do not follow a single socioeconomic gradient, with some risks concentrated among poorer groups and others more common among wealthier groups [6,7]. In African and South African settings, these gradients are influenced by changing food environments, urbanisation, unequal access to healthcare, education, employment opportunities, and differential exposure to health-promoting resources [7,8].

In South Africa, diabetes and hypertension remain major public health concerns and are patterned by socioeconomic and demographic factors [9–11]. Recent evidence also shows that self-reported diabetes may reflect unequal access to screening, diagnosis, and disease awareness across socioeconomic groups [12,13]. Broader evidence links diabetes burden to metabolic and social determinants, including obesity and access to prevention and care [14,15]. Tobacco use and harmful alcohol consumption continue to contribute to the burden of NCDs and vary across sex, education, employment, and socioeconomic groups [16–18]. Low fruit and vegetable intake, high salt intake, and physical inactivity are common modifiable risk factors associated with poor cardio metabolic outcomes [19–21]. Given the limited recent evidence from rural surveillance-linked settings, this study investigated factors associated with socioeconomic-related inequalities in the prevalence of chronic NCD risk factors among adults attending primary healthcare facilities within the DIMAMO Health and Demographic Surveillance System (HDSS) catchment area in rural South Africa.

## 2. Materials and methods

### 2.1. Ethics statement

The study was conducted in compliance with the principles outlined in the Declaration of Helsinki. Ethical approval was obtained from the Turfloop Research Ethics Committee (TREC), University of Limpopo (project number TREC/1692/2023: PG). Additional permissions were secured from the Limpopo Department of Health and the DIMAMO HDSS management. Written informed consent was obtained from all participants after a full explanation of the study purpose, procedures, and confidentiality assurances. Participation was voluntary, and respondents could withdraw at any time without consequence

### 2.2. Study design and setting

The study setting was the DIMAMO HDSS in the Capricorn District of Limpopo province, South Africa. The site was established in 1995, with a population of 8,000 individuals, then expanded to 42,000 individuals in 2010, and expanded again in 2018 to approximately 100,000 individuals. The DIMAMO HDSS is divided into three functional community areas (FCAs) and comprises 51 rural and semi-rural villages [22]. The site is serviced by eleven Primary Health Care (PHC) clinics, with one tertiary hospital approximately 10–30 km from the DIMAMO HDSS site [22,23]. The residents of these FCAs are mainly Northern Sotho-speaking [22].

### 2.3. Study population

The study included adults aged 18 years and older attending all PHC facilities located within the DIMAMO HDSS catchment area between 21 August and 11 October 2024. Although the study was conducted within the geographic area covered by the DIMAMO HDSS, participants were recruited from PHC facilities rather than directly from the HDSS population. Consequently, the study represents a facility-based sample of community members seeking healthcare services within the DIMAMO HDSS area.

### 2.4. Sample size and sampling techniques

The sample size was calculated using the single population proportion formula: n = Z^2^P(1 − P)/d^2^, where Z = 1.96 for a 95% confidence level, P = 0.518 based on a previous South African study [24] and d = 0.05. The calculated minimum sample size was 384. After adjusting for an anticipated 10% non-response rate, the final target sample size was 422. A total of 398 participants completed the survey, representing 94.3% of the target sample size.

A systematic random sampling method was used to select the participants. Community members who presented to the clinic on the scheduled day of data collection formed the sampling frame. The total number of attendees on the data collection day (𝑁) and the desired sample size (𝑛) were used to calculate the sampling interval (𝑘) using the formula: k = N/n. A random starting point (r) between 1 and k was selected using a random number generator. Thereafter, every k^th^ person presenting to the clinic was invited to participate until the target sample size was achieved.

Community members presenting to participating PHC facilities on scheduled data collection days constituted the sampling frame. Therefore, the study sample was limited to healthcare users and may not be fully representative of the broader DIMAMO HDSS population. Individuals attending PHC facilities may differ from the general population with respect to health status, healthcare-seeking behaviour, diagnosis history, and socioeconomic characteristics.

### 2.5. Tools and measures

Data were collected using a structured questionnaire adapted from the WHO STEPwise approach to NCD surveillance (STEPS) and WHO Study on Global Ageing and Adult Health (SAGE) instruments. Socioeconomic status was assessed using an asset-based wealth index constructed using Principal Component Analysis (PCA), a method commonly employed in demographic and health surveys to measure long-term household socioeconomic position. The questionnaire captured socio-demographic characteristics, socioeconomic conditions, tobacco use, alcohol consumption, dietary behaviour, salt consumption practices, physical activity, sedentary behaviour, and self-reported chronic health conditions. The questionnaire was originally developed in English and translated into Sepedi, the predominant local language spoken within the DIMAMO HDSS catchment area. The translated version was reviewed by bilingual speakers familiar with the local context to ensure conceptual consistency and clarity.

### 2.6. Measurements

For tobacco use, respondents were asked whether they were currently smoking tobacco products, yes = 1 and no = 0. Participants who reported consuming alcohol during the previous 30 days were asked about the number of standard alcohol drinks consumed each day in the past seven days. Consistent with WHO STEPS guidance, a standard drink was defined as containing approximately 20g of pure alcohol. Harmful alcohol use was defined as consuming three or more standard drinks per day (approximately 60g of pure alcohol), a threshold commonly used to identify heavy or risky alcohol consumption associated with adverse health outcomes and increased non-communicable disease risk. Low fruit and vegetable intake was defined as consuming fewer than five servings of fruits and vegetables per day, consistent with WHO dietary recommendations.

Participants were asked about behaviour associated with high dietary salt consumption, including the frequency of adding salt to food at the table, adding salt during cooking, and the consumption of processed or packaged foods known to contain high levels of sodium. Participants who reported regularly adding salt to food or frequent consumption of salty processed foods were classified as having high salt intake. The measure was based on self-reported dietary practices and was used as a proxy indicator of excess salt consumption because objective measures, such as 24-hour urinary sodium excretion, were not available.

An adapted version of the WHO STEPS questionnaire for adults was used to assess participants self-reported physical activity. The questionnaire assessed physical activity done in the past 7 days in the domains of work and walking (includes at work and at home, walking to travel from place to place, and any other walking for recreation, sport, exercise, or leisure). The time spent on moderate and vigorous intensity activities was estimated in terms of frequency (days per week) and duration (minutes per day) taken in each of the two physical activity domains. Total physical activity was calculated in Metabolic Equivalent of Task (MET)-minutes/week by summing: (minutes of moderate-intensity activity × 4 METs) + (minutes of vigorous-intensity activity × 8 METs). Physical inactivity was defined as engaging in less than 600 MET minutes of total activity per week, in accordance with WHO guidelines.

Sedentary behaviour was measured as total sitting or reclining time (in minutes) reported per day, excluding sleeping hours. It was classified into three categories: low (<240 min/day), moderate (240–479 min/day), and high (≥480 min/day). High sedentary behaviour was also coded as a binary outcome (1 => 480 min/day, 0 = < 480 min/day) for regression analysis.

### 2.7. Data management and analysis

All NCD risk factors were dichotomised to indicate whether respondents reported or did not report any NCD risk factors. Socioeconomic status (SES) was assessed using a household wealth index derived solely from asset ownership, housing characteristics, access to basic services, and livestock ownership. Variables included ownership of durable household assets (television, refrigerator, motor vehicle, and telephone), housing characteristics (roofing and flooring materials, cooking fuel source), access to basic services (electricity, drinking water, and sanitation facilities), and livestock ownership.

Principal Component Analysis (PCA) was used to derive the household wealth index following established approaches for measuring socioeconomic status in population-based studies where reliable income data are not available. Asset variables were coded as binary or categorical variables as appropriate and included in the PCA. The first principal component, which explained the largest proportion of variation in household assets and living conditions, was retained as the wealth index score. Each participant was assigned a wealth score based on the PCA-derived factor loadings.

Households with complete information on the variables included in the PCA were retained in the analysis. The resulting wealth scores were ranked from lowest to highest and divided into quintiles representing relative socioeconomic position within the study population. Participants were subsequently classified into five SES categories (poorest, poor, middle, richer, and richest). The quintiles were based on the distribution of the PCA-derived wealth scores and were selected to create approximately equal socioeconomic groups for comparative analysis.

Prevalence ratios (PRs) were used to quantify associations between socioeconomic status and study outcomes. Crude prevalence ratios were estimated in univariable analyses, and adjusted prevalence ratios (aPRs) were estimated using modified Poisson regression models with robust standard errors. Age, sex, education level, occupation, and marital status were included as potential confounders based on conceptual and epidemiological considerations. Adjusted prevalence ratios and 95% confidence intervals (CIs) were reported.

All NCD risk factor outcomes were dichotomised. Socioeconomic status was the main exposure variable and was categorized into quintiles. Crude prevalence ratios were estimated using univariable modified Poisson regression models with robust standard errors, while adjusted prevalence ratios were estimated using multivariable modified Poisson regression models with robust standard errors. Separate models were fitted for each NCD risk factor outcome. Socioeconomic status, age, sex, education level, occupation, and marital status were included as covariates based on conceptual and epidemiological considerations rather than statistical significance alone. Adjusted prevalence ratios, 95% confidence intervals, and p-values were reported. All analyses were conducted using Stata version 19.5, and statistical significance was determined at an alpha level of 0.05. All data supporting the findings of this study are available in the Supporting Information file (S1 Data).

## 3. Results

### 3.1. Demographic characteristics of the participants

Table 1 presents the characteristics of study participants in the DIMAMO HDSS, stratified by socioeconomic status (SES) quintiles ranging from Q1 (poorest) to Q5 (richest). A total of 398 participants were included in the study. The median age of participants was 34.5 years (IQR: 27–46 years). The largest proportion of participants was aged 31–50 years (41.9%), followed by those aged 18–30 years (37.7%). Across SES quintiles, the distribution of age groups was relatively similar, although a higher proportion of older participants (≥51 years) was observed in the poorest quintile.

**Table 1:** Characteristics of the study participants in DIMAMO HDSS by SES quintile.

Characteristic	Total	SES Quintiles				
		Q1 (Poorest)	Q2 (Poor)	Q3 (Middle)	Q4 (Richer)	Q5 (Richest)
**Total participants, n (%)**	398 (100)	110 (27.6)	71 (17.8)	87 (21.9)	63 (15.8)	67 (16.8)
**Age group (Years)**						
Median (IQR), years	34.5 (27-46)	35.5 (28-52)	31 (24-40)	33 (27-41)	39 (28-47)	34 (27-47)
18-30	150 (37.7)	34 (30.9)	32 (45.1)	38 (43.7)	21 (33.3)	25 (37.3)
31-50	167 (41.9)	45 (40.9)	29 (40.8)	38 (43.7)	29 (46.0)	26 (32.8)
≥51	81 (20.3)	31 (28.2)	10 (14.1)	11 (12.6)	13 (20.6)	16 (23.9)
**Level of education**						
No formal education	7 (1.8)	1 (0.9)	1 (1.4)	1 (1.2)	2 (3.2)	2 (2.9)
Primary	37 (9.3)	17 (15.4)	3 (4.2)	5 (5.7)	6 (9.5)	6 (8.9)
Secondary	251 (63.1)	81 (73.6)	47 (66.2)	65 (74.7)	31 (49.2)	27 (40.3)
Tertiary	103 (25.9)	11 (10.0)	20 (28.2)	16 (18.4)	24 (38.1)	32 (47.8)
**Marital status**						
Single	276 (69.3)	76 (69.1)	59 (83.1)	68 (78.2)	35 (55.6)	38 (56.7)
Married	91 (22.9)	25 (22.7)	9 (12.7)	16 (18.4)	21 (33.3)	20 (29.8)
Divorced	18 (4.5)	7 (6.4)	3 (4.2)	2 (2.3)	1 (1.6)	5 (7.5)
Widowed	13 (3.3)	2 (1.8)	0 (0.0)	1 (1.2)	6 (9.5)	4 (5.9)
**Occupation**						
Unemployed	240 (60.3)	77 (70.0)	45 (63.4)	60 (64.0)	30 (47.2)	28 (41.8)
Employed	101 (25.4)	22 (20.0)	15 (21.1)	15 (21.1)	21 (33.3)	23 (34.3)
Pensioner	28 (7.0)	10 (9.1)	3 (4.2)	3 (4.2)	7 (11.1)	7 (10.4)
Student	29 (7.3)	1 (0.9)	8 (11.3)	8 (11.3)	5 (7.9)	9 (13.4)
**Low Fruit and vegetable intake**						
Yes	353 (88.7)	102 (92.7)	67 (94.4)	74 (85.1)	54 (85.7)	56 (83.6)
No	45 (11.3)	8 (7.3)	4 (5.6)	13 (14.9)	9 (14.3)	11 (16.4)
**Smoking any tobacco**						
Yes	64 (16.1)	24 (21.8)	12 (16.9)	14 (16.1)	7 (11.1)	7 (10.4)
No	334 (83.9)	86 (78.2)	59 (83.1)	73 (83.9)	56 (88.9)	60 (89.6)
**Harmful alcohol use**						
Yes	124 (31.2)	27 (24.6)	22 (31.0)	27 (31.0)	23 (36.5)	25 (37.3)
No	274 (68.8)	83 (75.2)	49 (69.0)	60 (61.0)	40 (63.5)	42 (63.0)
**Self-reported hypertension**						
Yes	88 (22.1)	26 (23.6)	12 (16.9)	17 (19.5)	16 (25.4)	17 (25.4)
No	310 (77.9)	84 (76.4)	59 (83.1)	70 (80.5)	47 (74.6)	50 (74.6)
**Self-reported diabetes**						
Yes	28 (7.0)	5 (4.6)	1 (1.4)	5 (5.8)	6 (9.5)	11 (16.4)
No	370 (93.0)	105 (95.4)	70 (98.6)	82 (94.2)	57 (90.5)	56 (83.6)
**Physical Inactivity**						
Yes	73 (18.7)	29 (26.4)	14 (19.7)	10 (11.5)	8 (12.7)	12 (17.9)
No	325 (81.7)	81 (73.6)	57 (80.3)	77 (88.5)	55 (87.3)	55 (82.1)

Most participants had attained secondary education (61.3%), while 25.9% had tertiary education. Educational attainment tended to improve across the SES gradient, with tertiary education being more common among participants in the richer and richest quintiles. Single participants constituted the largest group (69.3%), followed by married participants (22.9%). Married participants were more frequently represented in the richer SES quintiles. Unemployment was prevalent within the study population, affecting 60.3% of participants. The proportion of unemployed participants was highest in the poorest SES quintile and decreased progressively across wealthier quintiles.

Low fruit and vegetable intake was highly prevalent, affecting 88.7% of participants across all SES groups. Current tobacco use was reported by 16.1% of participants, while harmful alcohol use was reported by 31.2%. Self-reported hypertension affected 22.1% of participants and self-reported diabetes affected 7.0%. The prevalence of diabetes appeared to increase across SES quintiles, with the highest prevalence observed among participants in the richest quintile. Physical inactivity was reported by 18.3% of participants, with a greater proportion observed among participants in the poorest SES quintile compared with those in the middle, richer, and richest quintiles.

### 3.2. Factors associated with socioeconomic inequalities in the prevalence of hypertension

Table 2 presents factors associated with the prevalence of hypertension. Socioeconomic status was not significantly associated with hypertension after adjustment for potential confounders. Compared with participants in the poorest SES quintile, those in the poor (aPR = 1.04; 95% CI: 0.57–1.90), middle (aPR = 1.19; 95% CI: 0.71–1.99), richer (aPR = 1.14; 95% CI: 0.69–1.88), and richest (aPR = 1.14; 95% CI: 0.68–1.93) quintiles had similar prevalence of hypertension. Participants aged ≥50 years had a significantly higher prevalence of hypertension than those aged 18–30 years (aPR = 3.35; 95% CI: 1.63–6.90; p = 0.001). Pensioners also had a significantly higher prevalence of hypertension compared with unemployed participants (aPR = 1.75; 95% CI: 1.05–2.89; p = 0.031).

**Table 2:** Factors associated with hypertension among participants.

Variable	Hypertension		Crude PR (95% CI)	p-value	aPR (95% CI)	p-value
	Yes n (%)	No n (%)				
Hypertension	88 (22.1)	310 (77.9)				
**Socioeconomic quintile**						
Poorest	26 (23.6)	84 (76.4)	Reference		Reference	
Poor	12 (16.9)	59 (83.1)	0.72 (0.39-1.32)	0.286	1.04 (0.57-1.90)	0.909
Middle	17 (19.5)	70 (80.5)	0.83 (0.48-1.42)	0.493	1.19 (0.71-1.99)	0.512
Richer	16 (25.4)	47 (74.6)	1.07 (0.63-1.85)	0.795	1.14 (0.69-1.88)	0.614
Richest	17 (25.4)	50 (74.6)	1.07 (0.63-1.83)	0.794	1.14 (0.68-1.93)	0.616
**Age group**						
18-30 years	14 (9.3)	136 (90.7)	Reference		Reference	
31-50 years	31 (18.6)	136 (88.4)	**1.99 (1.10-3.60)**	**0.023**	1.58 (0.85-2.92)	0.145
≥50 years	43 (53.1)	38 (46.9)	**5.69 (3.32-9.76)**	**<0.001**	**3.35 (1.63-6.90)**	**0.001**
**Sex**						
Male	18 (24.3)	56 (75.7)	Reference		Reference	
Female	70 (21.6)	254 (78.4)	0.89 (0.56-1.40)	0.608	1.33 (0.85-2.07)	0.206
**Education level**						
No formal education	2 (28.6)	5 (71.4)	Reference		Reference	
Primary	17 (45.9)	20 (54.1)	1.61 (0.47-5.47)	0.447	2.05 (0.73-5.76)	0.173
Secondary	49 (19.5)	202 (80.5)	0.68 (0.21-2.27)	0.534	1.51 (0.54-4.17)	0.430
Tertiary	20 (19.4)	83 (80.6)	0.68 (0.20-2.34)	0.541	1.59 (0.55-4.61)	0.393
**Employment status**						
Unemployed/Other	41 (17.1)	199 (82.9)	Reference		Reference	
Employed	27 (26.7)	74 (73.3)	**1.56 (1.02-2.40)**	**0.040**	1.25 (0.80-1.93)	0.327
Pensioner	19 (67.9)	9 (32.1)	**3.97 (2.72-5.80)**	**<0.001**	**1.75 (1.05-2.89)**	**0.031**
Student	1 (3.4)	28 (96.6)	0.20 (0.03-1.42)	0.107	0.33 (0.05-2.39)	0.272
**Marital status**						
Single/Never married	41 (14.9)	235 (85.1)	Reference		Reference	
Married	34 (37.4)	57 (62.6)	**2.52 (1.71-3.71)**	**<0.001**	1.29 (0.79-2.13)	0.310
Divorced	8 (44.4)	10 (55.6)	**2.99 (1.66-5.39)**	**<0.001**	1.19 (0.66-2.14)	0.570
Widowed	5 (38.5)	8 (61.5)	**2.59 (1.23-5.45)**	**0.012**	1.35 (0.69-2.65)	0.377

### 3.3. Factors associated with socioeconomic inequalities in the prevalence of diabetes

In the crude analysis, participants in the richest SES quintile had a significantly higher prevalence of diabetes than those in the poorest quintile (PR = 3.61; 95% CI: 1.31–9.95; p = 0.013). Similarly, participants aged ≥50 years had a significantly higher prevalence of diabetes compared with those aged 18–30 years (PR = 9.88; 95% CI: 2.96–32.94; p < 0.001), while females had a lower prevalence than males (PR = 0.26; 95% CI: 0.13–0.53; p < 0.001). However, after adjustment for potential confounders, none of the SES categories remained statistically significant. Participants in the richest SES quintile had a three-fold higher prevalence of diabetes compared with the poorest quintile, although this association did not reach statistical significance (aPR = 3.08; 95% CI: 0.92–10.25; p = 0.067). The wide confidence intervals observed indicate limited statistical precision and the results should therefore be interpreted cautiously. (Table 3).

**Table 3:** Factors associated with diabetes among participants.

Variable	Diabetes		Crude PR (95% CI)	p-value	aPR (95% CI)	p-value
	Yes n (%)	No n (%)				
Diabetes	28 (7.1)	370 (92.9				
**Socioeconomic quintile**						
Poorest	5 (4.5)	105 (95.5)	*Reference*		*Reference*	
Poor	1 (1.4)	70 (98.6)	0.31 (0.04-2.60)	0.281	0.63 (0.07-5.29)	0.669
Middle	5 (5.7)	82 (94.3)	1.26 (0.38-4.23)	0.704	2.50 (0.70-8.93)	0.159
Richer	6 (9.5)	57 (90.5)	2.10 (0.67-6.59)	0.206	1.97 (0.64-6.03)	0.237
Richest	11 (16.4)	57 (83.6)	**3.61 (1.31-9.95)**	**0.013**	3.08 (0.92-10.25)	0.067
**Age group**						
18-30 years	3 (2.0)	147 (98.0)	*Reference*		*Reference*	
31-50 years	9 (5.4)	158 (94.6)	2.69 (0.74-9.78)	0.132	1.64 (0.43-6.28)	0.469
≥50 years	16 (19.8)	65 (80.2)	**9.88 (2.96-32.94)**	**<0.001**	2.02 (0.35-11.49)	0.430
**Sex**						
Male	13 (17.6)	61 (82.4)	*Reference*		*Reference*	
Female	15 (4.6)	309 (95.4)	**0.26 (0.13-0.53)**	**<0.001**	0.53 (0.23-1.20)	0.129
**Education level**						
No formal education	2 (28.6)	5 (71.4)	*Reference*		*Reference*	
Primary	10 (27.0)	27 (73.0)	0.95 (0.26-3.43)	0.933	1.15 (0.27-4.90)	0.851
Secondary	8 (3.2)	243 (96.8)	**0.11 (0.03-0.43)**	**0.002**	0.22 (0.04-1.24)	0.087
Tertiary	8 (7.8)	95 (92.2)	0.27 (0.07-1.05)	0.058	0.42 (0.07-2.52)	0.339
**Employment status**						
Unemployed	11 (4.6)	229 (95.4)	*Reference*		*Reference*	
Employed	7 (6.9)	94 (93.1)	1.51 (0.60-3.79)	0.378	0.84 (0.33-2.15)	0.714
Pensioner	10 (35.7)	18 (64.3)	**7.79 (3.63-16.70)**	**<0.001**	2.47 (0.85-7.18)	0.097
Student	–	29 (100)	–	–	–	–
**Marital status**						
Never married	10 (3.6)	266 (96.4)	*Reference*		*Reference*	
Married	9 (9.9)	82 (90.1)	2.73 (1.14-6.51)	0.024	0.96 (0.36-2.56)	0.930
Divorced	5 (27.8)	13 (72.2)	**7.67 (2.93-20.08)**	**<0.001**	1.31 (0.43-4.03)	0.633
Widowed	4 (30.8)	9 (69.2)	**8.49 (3.07-23.52)**	**<0.001**	2.12 (0.64-7.06)	0.221

### 3.4. Factors associated with socioeconomic inequalities in the prevalence of current tobacco use

After adjustment, participants in the richer (aPR = 0.48; 95% CI: 0.23–1.03; p = 0.060) and richest (aPR = 0.45; 95% CI: 0.20–1.01; p = 0.054) SES quintiles had a lower prevalence of current tobacco use compared with those in the poorest quintile, although these associations were of borderline statistical significance. Female participants had a significantly lower prevalence of current tobacco use than males (aPR = 0.24; 95% CI: 0.16–0.38; p < 0.001). Participants with secondary education (aPR = 0.47; 95% CI: 0.25–0.88; p = 0.019) and tertiary education (aPR = 0.41; 95% CI: 0.18–0.94; p = 0.035) were also less likely to report current tobacco use compared with those with no formal education (Table 4).

**Table 4:** Factors associated with current tobacco use among participants.

Variable	Smoker		Crude PR (95% CI)	p-value	aPR (95% CI)	p-value
	Yes n (%)	No n (%)				
Current smoker	64 (16.1)	334 (83.9)				
**Socioeconomic quintile**						
Poorest	24 (21.8)	86 (78.2)	*Reference*		*Reference*	
Poor	12 (16.9)	59 (83.1)	0.77 (0.41-1.45)	0.424	1.07 (0.55-2.09)	0.842
Middle	14 (16.1)	73 (83.9)	0.74 (0.41-1.34)	0.317	0.93 (0.52-1.65)	0.804
Richer	7 (11.1)	56 (88.9)	0.51 (0.23-1.12)	0.092	0.48 (0.23-1.03)	0.060
Richest	7 (10.4)	60 (89.6)	0.48 (0.22-1.05)	0.066	0.45 (0.20-1.01)	0.054
**Age group**						
18-30 years	14 (9.3)	136 (90.7)	*Reference*		*Reference*	
31-50 years	26 (15.6)	141 (84.4)	1.67 (0.90-3.08)	0.101	1.44 (0.76-2.72)	0.262
≥50 years	24 (29.6)	57 (70.4)	**3.17 (1.74-5.80)**	**<0.001**	2.04 (0.86-4.84)	0.105
**Sex**						
Male	32 (43.2)	42 (56.8)	*Reference*		*Reference*	
Female	32 (9.9)	292 (90.1)	**0.23 (0.15-0.35)**	**<0.001**	**0.24 (0.16-0.38)**	**<0.001**
**Education level**						
No formal education	3 (42.9)	4 (57.1)	*Reference*		*Reference*	
Primary	11 (29.7)	26 (70.3)	0.69 (0.26-1.87)	0.469	0.68 (0.30-1.56)	0.368
Secondary	39 (15.5)	212 (84.5)	**0.36 (0.15-0.90)**	**0.028**	**0.47 (0.25-0.88)**	**0.019**
Tertiary	11 (10.7)	92 (89.3)	**0.25 (0.09-0.69)**	**0.008**	**0.41 (0.18-0.94)**	**0.035**
**Employment status**						
Unemployed	35 (14.6)	205 (85.4)	*Reference*		*Reference*	
Employed	16 (15.8)	85 (84.2)	1.09 (0.63-1.87)	0.766	0.94 (0.54-1.63)	0.821
Pensioner	10 (35.7)	18 (64.3)	**2.45 (1.37-4.39)**	**0.003**	1.26 (0.61-2.63)	0.532
Student	3 (10.3)	26 (89.7)	0.71 (0.23-2.16)	0.546	1.14 (0.37-3.48)	0.819
**Marital status**						
Never married	37 (13.4)	239 (86.6)	*Reference*		*Reference*	
Married	22 (24.2)	69 (75.8)	**1.80 (1.12-2.89)**	**0.014**	1.02 (0.53-1.97)	0.947
Divorced	2 (11.1)	16 (88.9)	0.83 (0.22-3.17)	0.784	**0.32 (0.11-0.99)**	**0.048**
Widowed	3 (23.1)	10 (76.9)	1.72 (0.61-4.86)	0.305	1.03 (0.36-2.96)	0.963

### 3.5. Factors associated with socioeconomic inequalities in the prevalence of harmful alcohol use

Table 5 presents factors associated with harmful alcohol use. After adjustment, participants in the richer (aPR = 1.58; 95% CI: 1.03–2.41; p = 0.035) and richest (aPR = 1.78; 95% CI: 1.11–2.85; p = 0.017) SES quintiles had a significantly higher prevalence of harmful alcohol use compared with participants in the poorest quintile. Female participants had a significantly lower prevalence of harmful alcohol use than males (aPR = 0.50; 95% CI: 0.37–0.69; p < 0.001). Similarly, employed participants had a lower prevalence of harmful alcohol use compared with unemployed participants (aPR = 0.62; 95% CI: 0.43–0.91; p = 0.015).

**Table 5:** Factors associated with harmful alcohol use.

Variable	Alcohol Intake		Crude PR (95% CI)	p-value	aPR (95% CI)	p-value
	Yes n (%)	No n (%)				
Alcohol intake	124 (31.2)	274 (68.8)				
**Socioeconomic quintile**						
Poorest	27 (24.5)	83 (75.5)	*Reference*		*Reference*	
Poor	22 (31.0)	49 (69.0)	1.26 (0.78-2.04)	0.339	1.32 (0.83-2.11)	0.241
Middle	27 (31.0)	60 (69.0)	1.26 (0.80-1.99)	0.311	1.21 (0.78-1.90)	0.392
Richer	23 (36.5)	40 (63.5)	1.49 (0.94-2.36)	0.093	**1.58 (1.03-2.41)**	**0.035**
Richest	25 (37.3)	42 (62.7)	1.52 (0.97-2.39)	0.069	**1.78 (1.11-2.85)**	**0.017**
**Age group**						
18-30 years	47 (31.3)	103 (68.7)	*Reference*		*Reference*	
31-50 years	61 (36.5)	106 (63.5)	1.17 (0.85-1.59)	0.333	1.16 (0.83-1.61)	0.382
≥50 years	16 (19.8)	65 (80.2)	0.63 (0.38-1.04)	0.070	0.74 (0.39-1.40)	0.349
**Sex**						
Male	35 (47.3)	39 (52.7)	*Reference*		*Reference*	
Female	89 (27.5)	235 (72.5)	**0.58 (0.43-0.78)**	**<0.001**	**0.50 (0.37-0.69)**	**<0.001**
**Education level**						
No formal education	2 (28.6)	5 (71.4)	*Reference*		*Reference*	
Primary	7 (18.9)	30 (81.1)	0.66 (0.17-2.55)	0.549	0.77 (0.19-3.14)	0.712
Secondary	85 (33.9)	166 (66.1)	1.19 (0.36-3.88)	0.779	1.07 (0.29-3.88)	0.922
Tertiary	30 (29.1)	73 (70.9)	1.02 (0.30-3.42)	0.975	0.84 (0.22-3.12)	0.791
**Employment status**						
Unemployed/Other	85 (35.4)	155 (64.6)	*Reference*		*Reference*	
Employed	25 (24.8)	76 (75.2)	0.70 (0.48-1.02)	0.065	**0.62 (0.43-0.91)**	**0.015**
Pensioner	6 (21.4)	22 (78.6)	0.61 (0.29-1.26)	0.178	0.70 (0.30-1.62)	0.405
Student	8 (27.6)	21 (72.4)	0.78 (0.42-1.44)	0.426	0.69 (0.35-1.37)	0.288
**Marital status**						
Never married	92 (33.3)	184 (66.7)	*Reference*		*Reference*	
Married	23 (25.3)	68 (74.7)	0.76 (0.51-1.12)	0.166	0.84 (0.55-1.28)	0.423
Divorced	3 (16.7)	15 (83.3)	0.50 (0.18-1.43)	0.195	0.64 (0.23-1.82)	0.408
Widowed	6 (46.1)	7 (53.9)	1.38 (0.75-2.55)	0.297	1.17 (0.57-2.37)	0.673

### 3.6. Factors associated with socioeconomic inequalities in the prevalence of high salt intake

In the crude analysis, participants in the richest SES quintile had a significantly higher prevalence of high salt intake compared with those in the poorest quintile (PR = 1.28; 95% CI: 1.03–1.59; p = 0.027). However, after adjustment for potential confounders, this association was attenuated and no longer statistically significant (aPR = 1.25; 95% CI: 0.99–1.56; p = 0.057). Likewise, the lower prevalence of high salt intake among participants aged ≥50 years observed in the crude analysis was no longer statistically significant after adjustment (aPR = 0.68; 95% CI: 0.46–1.01; p = 0.057). No other factors were significantly associated with high salt intake in the adjusted analysis (Table 6).

**Table 6:** Factors associated with high salt intake.

Variable	High Salt Intake		Crude PR (95% CI)	p-value	aPR (95% CI)	p-value
	Yes n (%)	No n (%)				
High salt intake	251 (63.1)	147 (36.9)				
**Socioeconomic quintile**						
Poorest	63 (57.3)	47 (42.7)	*Reference*		*Reference*	
Poor	40 (56.3)	31 (43.7)	0.98 (0.76-1.28)	0.902	0.91 (0.71-1.18)	0.478
Middle	58 (66.7)	29 (33.3)	1.16 (0.93-1.45)	0.175	1.10 (0.89-1.36)	0.399
Richer	41 (65.1)	22 (34.9)	1.14 (0.89-1.45)	0.302	1.12 (0.87-1.44)	0.373
Richest	49 (73.1)	18 (26.9)	**1.28 (1.03-1.59)**	**0.027**	1.25 (0.99-1.56)	0.057
**Age group**						
18-30 years	102 (68.0)	48 (32.0)	*Reference*		*Reference*	
31-50 years	115 (68.9)	52 (31.1)	1.01 (0.87-1.18)	0.869	1.05 (0.89-1.24)	0.533
≥50 years	34 (42.0)	47 (58.0)	**0.62 (0.47-0.82)**	**0.001**	0.68 (0.46-1.01)	0.057
**Sex**						
Male	42 (56.8)	32 (43.2)	*Reference*		*Reference*	
Female	209 (64.5)	115 (35.5)	1.14 (0.92-1.41)	0.243	1.07 (0.87-1.33)	0.524
**Education level**						
No formal education	4 (57.1)	3 (42.9)	*Reference*		*Reference*	
Primary	20 (54.1)	17 (45.9)	0.95 (0.47-1.92)	0.878	0.88 (0.46-1.69)	0.707
Secondary	153 (61.0)	98 (39.0)	1.07 (0.56-2.04)	0.846	0.83 (0.45-1.53)	0.551
Tertiary	74 (71.8)	29 (28.2)	1.26 (0.65-2.42)	0.492	0.92 (0.49-1.71)	0.784
**Employment status**						
Unemployed	162 (67.5)	78 (32.5)	*Reference*		*Reference*	
Employed	56 (55.5)	45 (44.6)	**0.82 (0.68-1.00)**	**0.049**	0.83 (0.68-1.01)	0.062
Pensioner	11 (39.3)	17 (60.7)	**0.58 (0.36-0.93)**	**0.024**	0.79 (0.46-1.36)	0.402
Student	22 (75.9)	7 (24.1)	1.12 (0.90-1.41)	0.306	1.03 (0.79-1.33)	0.839
**Marital status**						
Never married	185 (67.0)	91 (33.0	*Reference*		*Reference*	
Married	45 (49.4)	46 (50.6)	**0.74 (0.59-0.92)**	**0.008**	0.88 (0.70-1.11)	0.288
Divorced	11 (61.1)	7 (38.9)	0.91 (0.62-1.33)	0.632	1.25 (0.77-2.03)	0.365
Widowed	10 (76.9)	3 (23.1)	1.15 (0.84-1.56)	0.383	1.25 (0.88-1.78)	0.216

### 3.7. Factors associated with socioeconomic inequalities in the prevalence of low fruit and vegetable intake

Table 7 presents factors associated with low fruit and vegetable intake. After adjustment, participants aged 31–50 years had a higher prevalence of low fruit and vegetable intake compared with those aged 18–30 years (aPR = 1.09; 95% CI: 1.00–1.19; p = 0.038). Educational attainment was significantly associated with fruit and vegetable consumption. Participants with primary education (aPR = 0.84; 95% CI: 0.72–0.97; p = 0.020), secondary education (aPR = 0.89; 95% CI: 0.80–1.00; p = 0.045), and tertiary education (aPR = 0.79; 95% CI: 0.69–0.91; p = 0.001) had a lower prevalence of low fruit and vegetable intake compared with those with no formal education. Students also had a slightly higher prevalence of low fruit and vegetable intake compared with unemployed participants (aPR = 1.13; 95% CI: 1.00–1.28; p = 0.049).

**Table 7:** Factors associated with low fruit & vegetable intake.

Variable	Low Fruit & Veg Intake		Crude PR (95% CI)	p-value	aPR (95% CI)	p-value
	Yes n (%)	No n (%)				
Low fruit and vegetable intake	353 (88.7)	45 (11,3)				
**Socioeconomic quintile**						
Poorest	102 (92.7)	8 (7.3)	*Reference*		*Reference*	
Poor	67 (94.4)	4 (5.6)	1.02 (0.94-1.10)	0.657	1.02 (0.94-1.11)	0.635
Middle	74 (85.1)	13 (14.9)	0.92 (0.83-1.02)	0.099	0.92 (0.83-1.02)	0.125
Richer	54 (85.7)	9 (14.3)	0.92 (0.83-1.04)	0.175	0.96 (0.86-1.08)	0.516
Richest	56 (83.6)	11 (16.4)	0.90 (0.80-1.01)	0.086	0.94 (0.83-1.05)	0.277
**Age group**						
18-30 years	130 (86.7)	20 (13.3)	*Reference*		*Reference*	
31-50 years	151 (90.4)	16 (9.6)	1.04 (0.96-1.13)	0.299	**1.09 (1.00-1.19)**	**0.038**
≥50 years	72 (88.9)	9 (11.1)	1.03 (0.93-1.13)	0.618	1.05 (0.90-1.23)	0.523
**Sex**						
Male	62 (83.8)	12 (16.2)	*Reference*		*Reference*	
Female	291 (89.8)	33 (10.2)	1.07 (0.96-1.19)	0.202	1.07 (0.96-1.19)	0.204
**Education level**						
No formal education	7 (100)	–	*Reference*		*Reference*	
Primary	32 (86.5)	5 (13.5)	**0.86 (0.76-0.98)**	**0.026**	**0.84 (0.72-0.97)**	**0.020**
Secondary	230 (91.6)	21 (8.4)	**0.92 (0.88-0.95)**	**<0.001**	**0.89 (0.80-1.00)**	**0.045**
Tertiary	84 (81.5)	19 (18.5)	**0.82 (0.74-0.89)**	**<0.001**	**0.79 (0.69-0.91)**	**0.001**
**Employment status**						
Unemployed	216 (90.0)	24 (10.0)	*Reference*		*Reference*	
Employed	84 (83.2)	17 (16.8)	0.92 (0.84-1.02)	0.112	0.96 (0.87-1.05)	0.369
Pensioner	26 (92.3)	2 (7.1)	1.03 (0.92-1.15)	0.582	1.07 (0.91-1.25)	0.401
Student	27 (93.1)	2 (6.9)	1.03 (0.93-1.15)	0.538	**1.13 (1.00-1.28)**	**0.049**
**Marital status**						
Never married	249 (90.2)	27 (9.8)	*Reference*		*Reference*	
Married	75 (82.4)	16 (17.6)	0.91 (0.82-1.01)	0.084	0.92 (0.82-1.03)	0.144
Divorced	18 (100)	0	**1.11 (1.07-1.15)**	**<0.001**	1.09 (0.80-1.21)	0.113
Widowed	11 (84.6)	2 (15.4)	0.94 (0.74-1.19)	0.593	0.99 (0.77-1.28)	0.956

### 3.8. Factors associated with socioeconomic inequalities in the prevalence of physical inactivity

Table 8 presents factors associated with physical inactivity. After adjustment, participants in the middle (aPR = 0.44; 95% CI: 0.23–0.84; p = 0.013), richer (aPR = 0.35; 95% CI: 0.16–0.78; p = 0.010), and richest (aPR = 0.50; 95% CI: 0.27–0.92; p = 0.026) SES quintiles had a significantly lower prevalence of physical inactivity compared with those in the poorest quintile. Educational attainment was also associated with physical inactivity, with participants who had secondary (aPR = 0.27; 95% CI: 0.10–0.71; p = 0.008) and tertiary education (aPR = 0.34; 95% CI: 0.12–0.96; p = 0.043) having a lower prevalence of physical inactivity than those with no formal education. Pensioners had a significantly higher prevalence of physical inactivity compared with unemployed participants (aPR = 2.65; 95% CI: 1.13–6.19; p = 0.025).

**Table 8:** Factors associated with physical inactivity.

Variable	Physical inactivity		Crude PR (95% CI)	p-value	aPR (95% CI)	p-value
	Yes n (%)	No n (%)				
Physical inactivity	73 (18.3)	325 (81.7)				
**Socioeconomic quintile**						
Poorest	29 (26.4)	81 (73.6)	*Reference*		*Reference*	
Poor	14 (19.7)	57 (80.3)	0.75 (0.43-1.32)	0.313	0.70 (0.40-1.25)	0.230
Middle	10 (11.5)	77 (88.5)	**0.44 (0.22-0.85)**	**0.014**	**0.44 (0.23-0.84)**	**0.013**
Richer	8 (12.7)	55 (87.3)	**0.48 (0.23-0.99)**	**0.047**	**0.35 (0.16-0.78)**	**0.010**
Richest	12 (17.9)	55 (82.1)	0.68 (0.37-1.24)	0.207	**0.50 (0.27-0.92)**	**0.026**
**Age group**						
18-30 years	28 (18.7)	122 (81.3)	*Reference*		*Reference*	
31-50 years	26 (15.6)	141 (84.4)	0.83 (0.51-1.36)	0.465	0.83 (0.49-1.41)	0.491
≥50 years	19 (23.5)	62 (76.5)	1.26 (0.75-2.11)	0.386	0.59 (0.26-1.35)	0.212
**Sex**						
Male	19 (25.7)	55 (74.3)	*Reference*		*Reference*	
Female	54 (16.7)	270 (83.3)	0.65 (0.41-1.03)	0.065	0.76 (0.49-1.20)	0.245
**Education level**						
No formal education	3 (42.9)	4 (57.1)	*Reference*		*Reference*	
Primary	10 (27.0)	27 (73.0)	0.63 (0.23-1.73)	0.370	0.43 (0.15-1.22)	0.114
Secondary	40 (15.9)	211 (84.1)	**0.37 (0.15-0.92)**	**0.032**	**0.27 (0.10-0.71)**	**0.008**
Tertiary	20 (19.4)	83 (80.6)	0.45 (0.18-1.16)	0.100	**0.34 (0.12-0.96)**	**0.043**
**Employment status**						
Unemployed	37 (15.4)	203 (84.6)	*Reference*		*Reference*	
Employed	18 (17.8)	83 (82.2)	1.16 (0.69-1.93)	0.580	1.29 (0.76-2.20)	0.349
Pensioner	10 (35.2)	18 (64.3)	**2.32 (1.30-4.13)**	**0.004**	**2.65 (1.13-6.19)**	**0.025**
Student	8 (27.6)	21 (72.4)	1.79 (0.92-3.46)	0.084	1.88 (0.89-4.01)	0.100
**Marital status**						
Never married	48 (17.4)	228 (82.6)	*Reference*		*Reference*	
Married	15 (16.5)	76 (83.5)	0.95 (0.56-1.61)	0.843	0.95 (0.52-1.74)	0.874
Divorced	6 (33.3)	12 (66.7)	1.92 (0.95-3.87)	0.070	1.38 (0.64-3.00)	0.414
Widowed	4 (30.8)	9 (69.2)	1.77 (0.75-4.16)	0.191	1.96 (0.75-5.13)	0.171

## 4. Discussion

This study examined socioeconomic and demographic factors associated with the prevalence of chronic NCD risk factors among adults attending primary healthcare facilities within the DIMAMO HDSS catchment area. The findings reveal socioeconomic inequalities across behavioural and metabolic NCD risk factors, although the direction and magnitude of these associations differed across outcomes [1,5,7]. Age, sex, educational attainment, employment status, and socioeconomic position were associated with several NCD risk factors, highlighting the complex interplay between social and behavioural determinants of health in rural South Africa [6,7].

The study population was predominantly female. This pattern may reflect differential healthcare utilisation and participation in community-based health research, as women are generally more likely than men to access primary healthcare services and participate in health-related surveys in rural South Africa [8,11,12]. Therefore, the sex distribution observed in this study should not be interpreted solely as representative of the underlying population demographic structure. Significant sex differences were observed for current tobacco use and harmful alcohol use, with males exhibiting a higher prevalence of both risk factors. These findings are consistent with national and regional evidence demonstrating higher tobacco use and alcohol use among men than women in South Africa [16,25,26]. Similar patterns have been reported across several African settings, where men consistently exhibit a higher burden of this behavioural risk factors [1,17,18].

Hypertension was associated with age and pensioner status. Participants aged 50 years and older had a substantially higher prevalence of hypertension compared with younger adults, while pensioners were more likely to report hypertension than unemployed participants. These findings are consistent with the established relationship between ageing and cardiovascular risk and likely reflect the cumulative effects of long-term exposure to behavioural, metabolic, and physiological risk factors [27,28]. Despite socioeconomic differences in the prevalence of hypertension, no statistically significant association was observed between SES and hypertension after adjustment, suggesting that hypertension may affect individuals across the socioeconomic spectrum in rural communities [27,29].

Participants in the richest socioeconomic quintile demonstrated a higher prevalence of self-reported diabetes than those in the poorest quintile, although the association did not reach statistical significance after adjustment and should therefore be interpreted cautiously. Nevertheless, the observed pattern is consistent with the epidemiological and nutrition transition framework frequently reported in low- and middle-income countries [9,13]. Because diabetes was self-reported, differential access to screening, diagnosis, and disease awareness may partly explain the observed SES pattern rather than indicating true differences in underlying disease prevalence [13,30]. Individuals with higher socioeconomic status are more likely to undergo routine screening and receive a diagnosis, whereas diabetes may remain undiagnosed among poorer individuals. Consequently, the findings should be interpreted as associations and not as evidence of causal relationships or true differences in underlying disease prevalence.

Current tobacco use was less prevalent among participants with secondary and tertiary education and among females. Participants in the richer and richest SES quintiles also demonstrated a lower prevalence of current tobacco use than those in the poorest quintile, although these associations were of borderline statistical significance. These findings are consistent with evidence indicating that individuals with secondary or post-secondary education are less likely to smoke than those with no formal education, and that women report substantially lower smoking prevalence than men [16,26]. The lower prevalence of tobacco use among females likely reflects prevailing social and cultural norms in rural South African communities, where smoking among women remains less socially acceptable than among men.

Harmful alcohol use showed a different socioeconomic pattern. Participants in the richer and richest socioeconomic quintiles had a significantly higher prevalence of harmful alcohol use than participants in the poorest quintile. Higher socioeconomic status may increase access to commercial alcohol outlets and provide greater disposable income to purchase alcohol [17,31]. Furthermore, alcohol consumption among higher socioeconomic groups may be reinforced by social and occupational environments where alcohol use is more common. Female participants and employed individuals had a lower prevalence of harmful alcohol use than their counterparts. These findings support previous evidence that gender norms, employment responsibilities, and lifestyle factors influence drinking behaviour [18,31].

Although participants in the richest SES quintile demonstrated a higher prevalence of high salt intake than those in the poorest quintile, the association did not remain statistically significant after adjustment for potential confounders. Nevertheless, the observed pattern may suggest increasing consumption of processed and packaged foods among wealthier households undergoing dietary transition, but it should be interpreted cautiously because salt intake was self-reported [32,33]. Future studies utilizing biomarkers such as 24-hour urinary sodium excretion would provide more accurate estimates of salt intake and socioeconomic differences in dietary sodium consumption [34,35].

Educational attainment was significantly associated with fruit and vegetable consumption. Participants with primary, secondary, and tertiary education demonstrated a lower prevalence of low fruit and vegetable intake compared with participants who had no formal education. These findings suggest that education may improve dietary choices through increased nutrition knowledge, health literacy, and awareness of healthy eating practices [21,36]. However, despite these associations, low fruit and vegetable intake remained highly prevalent across all socioeconomic groups, indicating that factors such as food affordability, accessibility, and availability may continue to limit healthy dietary practices in rural communities [36–38].

Physical inactivity demonstrated a clear socioeconomic gradient. Participants in the middle, richer, and richest socioeconomic groups had a significantly lower prevalence of physical inactivity than participants in the poorest group. This finding suggests that individuals with greater socioeconomic resources may have more opportunities to engage in recreational physical activity and health-promoting behaviour [20,39]. Conversely, poverty may limit access to safe recreational spaces and opportunities for structured physical activity. Educational attainment was also associated with lower prevalence of physical inactivity, while pensioners had a significantly higher prevalence of physical inactivity. The higher prevalence of physical inactivity among pensioners likely reflects age-related declines in physical functioning and reduced participation in occupational and transport-related activity among older adults [19,39].

An important finding of this study is that socioeconomic status was not uniformly associated with all NCD risk factors. Higher socioeconomic status was associated with a higher prevalence of harmful alcohol use and a lower prevalence of physical inactivity, while a tendency towards a higher prevalence of diabetes and high salt intake was also observed [5,9]. Comparable LMIC and South African evidence indicates that socioeconomic advancement can increase some diet-related and metabolic risks while reducing other risks through improved education, health literacy, and access to health-promoting resources [18,31]. These patterns support the need to interpret socioeconomic gradients in NCD risk factors according to the specific behavioural or metabolic outcome under study [21,37].

This study has several notable strengths. Although conducted among adults attending primary healthcare facilities within the DIMAMO HDSS catchment area, it provides important evidence on socioeconomic inequalities in NCD risk factors in a rural South African setting where such data remain limited. The study assessed a broad range of behavioural and metabolic NCD risk factors using instruments adapted from standardized surveillance tools, improving comparability with other NCD risk factor studies. In addition, the use of a PCA-derived household wealth index and modified Poisson regression with robust standard errors enabled direct estimation of prevalence ratios, which are appropriate for cross-sectional binary outcomes and socioeconomic inequality analyses

The findings should be interpreted in light of several limitations. The cross-sectional design precludes conclusions regarding causality or temporal relationships. The study was facility-based and included individuals attending primary healthcare facilities within the DIMAMO HDSS catchment area; therefore, the findings may not be generalised to the broader population. Several outcomes, particularly self-reported diabetes, had relatively small numbers of events, resulting in wide confidence intervals and reduced statistical precision. Hypertension, diabetes, salt intake, and dietary behaviour were based on self-reported information and may therefore be subject to recall bias, reporting bias, and differential disease awareness across socioeconomic groups.

## 5. Conclusion

In conclusion, this study identified socioeconomic inequalities in the prevalence of selected non-communicable disease (NCD) risk factors among adults attending primary healthcare facilities within the DIMAMO HDSS catchment area. Higher socioeconomic status was associated with a higher prevalence of harmful alcohol use and a lower prevalence of physical inactivity, while participants in higher socioeconomic groups also demonstrated a tendency towards a higher prevalence of self-reported diabetes and high salt intake. In contrast, current tobacco use was less prevalent among individuals with higher educational attainment and those in wealthier socioeconomic groups. These findings highlight the complex and non-uniform nature of socioeconomic gradients in NCD risk factors in rural South Africa, reflecting the ongoing epidemiological and nutrition transition within the population.

Given the facility-based sample, cross-sectional study design, reliance on self-reported measures, and relatively modest sample size, the findings should be interpreted as associations rather than causal relationships. Nevertheless, the study provides valuable evidence that the distribution of NCD risk factors differs across socioeconomic groups and that socioeconomic position remains an important consideration in NCD prevention and control efforts. Tailored public health strategies that address the specific risk profiles of different socioeconomic groups may contribute to reducing the burden of NCDs and associated health inequalities in rural communities. Further population-based and longitudinal studies are needed to confirm these findings and to better understand the pathways through which socioeconomic factors influence NCD risk.

## Supporting information

S1 Data Dataset used in the analysis. (XLSX)
