# Supplementary material for: Factors associated with socioeconomic inequalities in the prevalence of chronic NCD risk factors in the DIMAMO surveillance site, South Africa
Source: PLOS Glob Public Health. 2026 Oct 26;6(10):e0007483. doi: 10.1371/journal.pgph.0007483 (PMC13704811; doi:10.1371/journal.pgph.0007483)
Supplement: Supplementary file 2 [file pgph.0007483.s002.docx]

Thank you for the opportunity to review this manuscript. The study addresses an important public health question. Overall, the manuscript has the potential to contribute to the literature on NCD risk factor distribution in rural LMIC settings. However, several methodological, analytical, and reporting issues should be addressed to improve the clarity and strength of the manuscript before publication.

**Major comments**

1. **Clarify study design and representativeness**

The manuscript describes the study as being conducted within the DIMAMO HDSS, but the methods indicate that participants were recruited from selected primary healthcare facilities, with the sampling frame limited to community members presenting to the clinics on data collection days. This should be clearly described as a facility-based sample within the DIMAMO HDSS area, unless the authors can justify that it represents the broader HDSS population. Because PHC attendees may differ from the general population in health status, healthcare-seeking behavior, diagnosis history, and socioeconomic profile, the implications for selection bias and generalizability should be discussed.

2. **Provide more detail on the wealth index**

SES is central to the study objective and conclusion, but the construction of the wealth index is not sufficiently clear. Please describe the exact approach used to derive the wealth score (for instance principal component analysis, equal weighting, summed scoring), how items were selected, how missing values were handled, and why the reported quintile cutoffs were chosen. Providing a methodological justification and relevant reference would strengthen reproducibility and interpretation.

3. **Address small event counts and statistical precision**

Some outcomes had few events, like self-reported diabetes with only 28 cases. Given the number of covariates in the adjusted model and the wide confidence intervals, this should be added to the limitations, and the diabetes-related findings should be interpreted more cautiously.

4. **Clarify covariate selection and model-building strategy**

The methods section states that variables with p < 0.20 in univariate models were considered for multivariable models, while SES was retained regardless of significance. Please clarify whether this was based on the “purposeful selection approach” and provide an appropriate reference for the screening threshold. It is also not clear whether the same covariate set was used across all outcomes or whether each outcome had a separate model-building process. Please clarify how final covariates were selected for each adjusted model and whether selection was based only on statistical criteria or also informed by conceptual/confounding considerations.

5. **Interpret cross-sectional associations more cautiously**

Given the cross-sectional design, the manuscript cannot establish temporality or causality. The authors should avoid causal language such as “drivers,” or “determinants” unless carefully qualified. I suggest reframing the title, objective, discussion, and conclusion to use terms such as “factors associated with” or “correlates of” socioeconomic inequalities in NCD risk factors. Findings should be interpreted as associations only.

6. **Self-reported hypertension and diabetes may introduce detection bias**

Hypertension and diabetes were self-reported. This is an important limitation in an SES analysis, because higher-SES participants may have better access to screening and may therefore be more likely to know and report a diagnosis. The observed positive association between higher SES and diabetes may partly reflect differential diagnosis or awareness rather than true disease prevalence. This should be discussed more explicitly.

7. **Discussion could be strengthened conceptually**

The discussion appropriately compares findings with prior literature, but it could be more analytical regarding the mixed SES patterns observed. For example, higher SES was associated with higher odds of diabetes, harmful alcohol use, and high salt intake, but lower odds of smoking and physical inactivity. While a lower SES was associated with smoking and physical inactivity. Therefore, the SES gradient is not uniform across all NCD risk factors. A brief explanation of why higher SES may be associated with some risks but appear protective for others would strengthen the interpretation and help contextualize the findings in rural South Africa.

8. **Avoid overgeneralized policy recommendations**

The conclusion recommends targeted interventions by SES group. While this is reasonable, the recommendations should be tempered given the facility-based sample, cross-sectional design, self-reported measures, and limited sample size. The authors should present these as potential implications rather than definitive intervention priorities.

**Minor comments**

1. Please correct typographical errors, including “fruit ang vegetable intake.”
2. Please ensure consistency in terminology. The manuscript uses “poor physical activity,” “physical inactivity,” “smoking,” and “harmful smoking.” It would be clearer to use consistent labels throughout.
3. The alcohol use definition would benefit from clarification. The manuscript defines excessive drinking as three or more drinks per day, approximately 60 g of alcohol. Please explain how this definition aligns with WHO STEPS or other established thresholds.
4. Please clarify whether “low fruit intake” in the results and Table 1 refers to fruit only or combined fruit and vegetable intake, since other sections refer to low fruit and vegetable intake. Also please make sure the terminology is consistent (low or poor intake)
