# Supplementary material for: Factors associated with socioeconomic inequalities in the prevalence of chronic NCD risk factors in the DIMAMO surveillance site, South Africa
Source: PLOS Glob Public Health. 2026 Oct 26;6(10):e0007483. doi: 10.1371/journal.pgph.0007483 (PMC13704811; doi:10.1371/journal.pgph.0007483)
Supplement: Supplementary file 10002 [file pgph.0007483.s003.pdf]

Editorial Office

PLOS Global Public Health,

Subject: Clarification on Data Collection Dates

Dear Editor,

Thank you for your careful review and the helpful feedback.

I would like to clarify that the participant recruitment and data collection for this study were conducted between 21 August and 11 October 2024, not 2025. I inadvertently entered the year incorrectly in the submission materials, and I sincerely apologise for this oversight.

Given this correction, the ethics approval obtained for the study covers the relevant 2024 period. I am happy to provide any documentation you may require to verify the 2024 data collection, including:

- Fieldwork schedules and recruitment logs dated in 2024
- System-generated timestamps/exports showing 2024 entries

Please let me know if you would like me to upload a brief clarification note in the resubmission and attach the above evidence. If there are any specific formats or forms you prefer, I will gladly follow your guidance.

Thank you again, and I appreciate your consideration.

Sincerely**,**

Khuliso Ravhuhali
