# Supplementary material for: Factors associated with socioeconomic inequalities in the prevalence of chronic NCD risk factors in the DIMAMO surveillance site, South Africa
Source: PLOS Glob Public Health. 2026 Oct 26;6(10):e0007483. doi: 10.1371/journal.pgph.0007483 (PMC13704811; doi:10.1371/journal.pgph.0007483)
Supplement: Supplementary file 2 [file pgph.0007483.s004.docx]

**Response to the Editor and Reviewers**

**Manuscript ID:** *PGPH-D-25-03760*

**Title:** *Determinants of socioeconomic inequalities in the prevalence of chronic NCDs risk factors in the DIMAMO surveillance site, South Africa*

Dear Editor and Reviewers,

We thank the Editor and the Reviewers for their careful review of our manuscript and for the constructive comments provided. We have revised the manuscript accordingly. All changes have been incorporated into the revised manuscript and are highlighted in track changes. Detailed responses to each comment are provided below.

**Reviewer 1**

**Comment 1: Analysis**

This is an inequality analysis. Typically, in such analyses, we use an absolute measure of association (e.g., a proportion or prevalence difference). And if we use a relative measure of association, we use proportion ratios (or prevalence ratios). The odds ratio, as the authors chose here, is not the recommended measure of association for exploring inequality. Please reconsider this choice. I would suggest presenting unadjusted absolute differences and replacing the OR with proportion ratios (PR).

**Response:**

We agree that prevalence ratios are more appropriate for cross-sectional inequality analyses. Accordingly, we replaced logistic regression models with modified Poisson regression models using robust standard errors and report adjusted prevalence ratios (aPRs) with 95% confidence intervals. Separate multivariable models were retained for each NCD risk factor outcome to evaluate associations with SES, age, sex, education, occupation, and marital status.

**Comment 2: Covariate selection**

Please do not use p-values to consider what should be adjusted for. Confounding is not about p-values, and you have only 6 covariates. Gender, age, education, and socioeconomic status should be part of the minimal set of covariates to keep.

**Response:**

We thank the reviewer for this important comment and agree that confounder selection should not be based solely on p-values. We have revised the Methods section to clarify that the multivariable models were informed primarily by conceptual and epidemiological considerations. Specifically, socioeconomic status, age, sex, education, occupation, and marital status were considered a priori potential confounders based on previous literature and were retained in adjusted analyses regardless of statistical significance. We have therefore removed the emphasis on p-value-based variable selection and clarified our model-building approach accordingly.

**Comment 3: Table 1**

- Present a row of totals
- Reconsider the age categories and add median and IQR
- Please revise the parentheses for the proportions in Alcohol consumption (eg: Q1 and Q2 columns)
- We do not need p-values on this table. But if you included, why did we miss the p-value for “Low fruit intake”? Please add below the table how these p-values were calculated.

**Response:**

- Thank you. A total row has been added to Table 1.
- We agree. The median age and interquartile range (IQR) have now been added to Table 1 and mentioned in the Results section.
- Thank you for noting this formatting error. The proportions have been corrected in Table 1.
- We agree that these p-values are not essential. They have been removed from Table 1 to improve it.

**Comment 4: Table 3**

Table 3: For categories with zeros, just put a dash. Not a number. This is for the employment status for students.

**Response:**

We agree. Zero-event cells have been replaced with dashes.

**Comment 5: Table 7**

Table 7: For categories with zeros, just put a dash. Not a number. This is for the education status for tertiary. And marital status for divorce.

**Response:**

Thank you. All cells with zero observations now display a dash instead of a numerical estimate.

**Comment 6: Abstract**

The same goes for the abstract. Be careful to speak of inequalities and use OR in the abstract.

**Response:**

Thank you. Following revisions to the analytical approach, the Abstract has been updated to use prevalence ratios and to consistently describe socioeconomic inequalities rather than odds.

**Reviewer 2**

**Comment 1: Data Collection Date Discrepancy**

The manuscript text (Section 2.2) states data collection occurred from 21 August to 11 October 2024, but the author’s note to the editor indicates this was erroneously listed as 2025 in submission materials. This must be explicitly corrected in the manuscript (Abstract, Methods, and any relevant footnotes) to maintain credibility. A brief statement in the Methods or as a footnote should clarify and confirm that ethics approval covered the correct period.

**Response:**

Thank you for identifying this inconsistency. The correct data collection period was 21 August to 11 October 2024. This has now been corrected throughout the manuscript. We have also clarified that the ethics approval covered this study period.

**Comment 2: Sample Size and Power Limitations**

The sample size (n=398) is modest, especially when stratified into SES quintiles and multiple risk factors. This likely limits the power to detect significant associations, particularly for less prevalent outcomes (e.g., diabetes n=28). The authors should:

Discuss this limitation more explicitly in the Discussion.

Consider presenting confidence intervals consistently and noting where wide CIs indicate uncertainty (e.g., diabetes aOR: 1.5–18.1).

Avoid overinterpreting non-significant findings.

**Response:**

We have expanded the limitations section to explicitly acknowledge that the sample size was relatively modest, particularly after stratification by SES quintiles and individual NCD risk factors. We now discuss the potential impact of limited statistical power and note that outcomes with few events, especially self-reported diabetes (n = 28), may have produced imprecise estimates, reflected by wide confidence intervals. We have also revised the discussion of diabetes findings to emphasize the uncertainty surrounding the magnitude of the observed association and have avoided overinterpreting non-significant findings throughout the manuscript by referring to them as "not statistically significant" rather than implying the absence of an association.

**Comment 3. Measurement and Bias**
Self-reported data for hypertension, diabetes, diet, and salt intake are subject to recall and social desirability bias. This should be highlighted as a key limitation.

**Response:**

We have expanded the limitations section to explicitly acknowledge that hypertension, diabetes, fruit and vegetable intake, and salt intake were based on self-reported information and are therefore subject to recall and social desirability bias. We further discuss the potential for differential diagnosis and disease awareness across socioeconomic groups, particularly for hypertension and diabetes, and note that these factors may have influenced the observed associations. The limitations related to self-reported dietary and salt intake measures have also been highlighted more explicitly.

**Comment 4: Wealth index construction**

Wealth index construction: The description is brief. Provide more detail on how the index was derived (PCA? simple sum?), which assets were included, and how quintiles were defined. This is crucial for replicability and interpretation.

**Response:**

We have expanded the Methods section to provide a detailed description of the wealth index construction. Specifically, we clarify that socioeconomic status was measured using an asset-based wealth index derived through Principal Component Analysis (PCA), describe the assets and household characteristics included, explain the handling of missing data, and clarify that participants were categorized into SES quintiles based on the distribution of PCA-derived wealth scores. We have also added methodological references commonly cited for PCA-based wealth index construction.

**Comment 5: Salt intake measurement**

Salt intake measurement: The method is not clearly described. How was high salt intake defined? If based on self-reported addition or consumption of processed foods, this should be explicitly stated and its limitations acknowledged.

**Response:**

We have clarified the assessment of high salt intake in the Methods section by explicitly describing the questionnaire items used to classify participants according to salt consumption behaviours, including salt added to food and consumption of salty processed foods. We also acknowledge that the measure was based on self-reported behaviour rather than objective sodium assessment. Furthermore, we expanded the Discussion and Limitations sections to discuss the potential for recall bias and misclassification and to note that future studies using objective measures such as 24-hour urinary sodium excretion would provide more accurate estimates of salt intake.

**Comment 6: Diabetes and higher SES**

The finding that higher SES is linked to higher diabetes risk is intriguing but should be contextualized within the epidemiological transition framework. The authors should more deeply discuss potential mechanisms (e.g., dietary shift, sedentary behavior) and contrast with patterns in high-income countries.

**Response:**

We have expanded the Discussion to interpret the positive association between higher SES and diabetes within the epidemiological transition framework. Specifically, we discuss how dietary shifts, increased consumption of processed foods, reduced occupational physical activity, and more sedentary lifestyles among higher-SES groups may contribute to increased diabetes risk in rural South Africa. We also contrast these findings with patterns commonly observed in high-income countries, where diabetes is more strongly concentrated among lower socioeconomic groups. In addition, we acknowledge that differential access to screening and diagnosis may partially explain the observed association because diabetes status was self-reported.

**Comment 7: Secondary education and low fruit/vegetable intake**

The association between secondary education and lower fruit/vegetable intake is counterintuitive and merits more nuanced discussion (e.g., education without economic empowerment, food environment constraints).

**Response:**

We agree that the observed association between secondary education and low fruit and vegetable intake warrants a more nuanced interpretation. We have expanded the Discussion to explain that educational attainment may not necessarily be accompanied by economic empowerment or improved access to healthy foods in rural settings. We also discuss how food environment constraints, affordability, unemployment, and limited availability of fruits and vegetables may contribute to this pattern. This additional discussion provides a more contextualized interpretation of the findings within the rural South African setting.

**Reviewer 3**

**Comment 1: Clarify study design and representativeness**
The manuscript describes the study as being conducted within the DIMAMO HDSS, but the methods indicate that participants were recruited from selected primary healthcare facilities, with the sampling frame limited to community members presenting to the clinics on data collection days. This should be clearly described as a facility-based sample within the DIMAMO HDSS area, unless the authors can justify that it represents the broader HDSS population. Because PHC attendees may differ from the general population in health status, healthcare-seeking behavior, diagnosis history, and socioeconomic profile, the implications for selection bias and generalizability should be discussed.

**Response:**

We agree that the study was conducted among adults attending primary healthcare facilities within the DIMAMO HDSS catchment area and therefore represents a facility-based sample rather than a population-based sample of the HDSS population. We have revised the Methods section to clearly describe the sampling frame and recruitment process. We have also revised the Discussion and Limitations sections to acknowledge the potential for selection bias arising from healthcare-seeking behaviour and differences in disease awareness, diagnosis history, and socioeconomic characteristics among PHC attendees. Furthermore, we have tempered statements regarding representativeness and generalizability and clarified that the findings may not be fully representative of the broader DIMAMO HDSS population.

**Comment 2: Provide more detail on the wealth index**
SES is central to the study objective and conclusion, but the construction of the wealth index is not sufficiently clear. Please describe the exact approach used to derive the wealth score (for instance principal component analysis, equal weighting, summed scoring), how items were selected, how missing values were handled, and why the reported quintile cutoffs were chosen. Providing a methodological justification and relevant reference would strengthen reproducibility and interpretation.

**Response:**

We have expanded the description of the wealth index construction to improve reproducibility and interpretation. Specifically, we now describe the variables included in the index, the scoring approach used to derive the composite wealth score, the handling of missing data, and the rationale for the SES categorization. We have also included references supporting the use of asset-based wealth indices as measures of socioeconomic status in population health studies. Furthermore, we clarify that the SES categories were derived from the observed distribution of wealth scores within the study population.

**Comment 3: Address small event counts and statistical precision**
Some outcomes had few events, like self-reported diabetes with only 28 cases. Given the number of covariates in the adjusted model and the wide confidence intervals, this should be added to the limitations, and the diabetes-related findings should be interpreted more cautiously.

**Response:**

We agree that the small number of diabetes cases (n = 28) may have limited statistical precision, particularly given the number of covariates considered in the adjusted model. We have expanded the limitations section to acknowledge the potential for unstable estimates and the wide confidence intervals observed for diabetes-related associations. We have also tempered the interpretation of the diabetes findings in the Discussion and emphasize that these results should be interpreted cautiously and confirmed in larger studies.

**Comment 4: Clarify covariate selection and model-building strategy**
The methods section states that variables with p < 0.20 in univariate models were considered for multivariable models, while SES was retained regardless of significance. Please clarify whether this was based on the “purposeful selection approach” and provide an appropriate reference for the screening threshold. It is also not clear whether the same covariate set was used across all outcomes or whether each outcome had a separate model-building process. Please clarify how final covariates were selected for each adjusted model and whether selection was based only on statistical criteria or also informed by conceptual/confounding considerations.

**Response:**

We have clarified the model-building strategy in the Methods section. The use of a p < 0.20 threshold was based on the purposeful selection approach described by Hosmer, Lemeshow and Sturdivant (2013), which recommends using a more liberal screening criterion to avoid excluding potentially important confounders during initial model development. We have also clarified that covariate selection was not based solely on statistical criteria but was additionally informed by conceptual and epidemiological considerations. SES was retained in all models as the primary exposure variable, while age, sex, education, occupation, and marital status were evaluated as potential confounders. Furthermore, we now explicitly state that separate multivariable models were developed for each NCD risk factor outcome and that the final covariate set could therefore differ across outcomes.

**Comment 5: Interpret cross-sectional associations more cautiously**
Given the cross-sectional design, the manuscript cannot establish temporality or causality. The authors should avoid causal language such as “drivers,” or “determinants” unless carefully qualified. I suggest reframing the title, objective, discussion, and conclusion to use terms such as “factors associated with” or “correlates of” socioeconomic inequalities in NCD risk factors. Findings should be interpreted as associations only.

**Response:**

We agree that the cross-sectional design does not permit causal inference or establishment of temporal relationships. We have revised the title, objectives, results headings, discussion, and conclusion to avoid causal language such as “determinants” and “drivers” and now consistently describe the findings as associations. We have also strengthened the limitations section to explicitly acknowledge that the observed relationships should be interpreted as associations rather than causal effects.

**Comment 6: Self-reported hypertension and diabetes may introduce detection bias**
Hypertension and diabetes were self-reported. This is an important limitation in an SES analysis, because higher-SES participants may have better access to screening and may therefore be more likely to know and report a diagnosis. The observed positive association between higher SES and diabetes may partly reflect differential diagnosis or awareness rather than true disease prevalence. This should be discussed more explicitly.

**Response:**

We agree that the use of self-reported diabetes and hypertension may introduce detection bias, particularly in the context of socioeconomic analyses. Individuals with higher socioeconomic status may have greater access to healthcare services and screening opportunities and may therefore be more likely to know and report a diagnosis. We have expanded the Discussion section to acknowledge that the positive association between higher SES and self-reported diabetes may partly reflect differential diagnosis and disease awareness rather than true differences in underlying prevalence.

**Comment 7. Discussion could be strengthened conceptually**
The discussion appropriately compares findings with prior literature, but it could be more analytical regarding the mixed SES patterns observed. For example, higher SES was associated with higher odds of diabetes, harmful alcohol use, and high salt intake, but lower odds of smoking and physical inactivity. While a lower SES was associated with smoking and physical inactivity. Therefore, the SES gradient is not uniform across all NCD risk factors. A brief explanation of why higher SES may be associated with some risks but appear protective for others would strengthen the interpretation and help contextualize the findings in rural South Africa.

**Response:**

We agree that the socioeconomic gradient was not consistent across all NCD risk factors and that a more nuanced interpretation was warranted. We have expanded the Discussion to explain how higher SES may increase exposure to certain behavioural and metabolic risk factors, such as harmful alcohol use, high salt intake, and diabetes, while simultaneously being associated with lower smoking prevalence and physical inactivity through mechanisms such as greater health awareness, access to health information, and lifestyle differences. We also contextualize these findings within the framework of the epidemiological and nutrition transition occurring in rural South Africa.

**Comment 8: Avoid overgeneralized policy recommendations**
The conclusion recommends targeted interventions by SES group. While this is reasonable, the recommendations should be tempered given the facility-based sample, cross-sectional design, self-reported measures, and limited sample size. The authors should present these as potential implications rather than definitive intervention priorities.

**Response:**

We agree that the study's facility-based sample, cross-sectional design, self-reported measures, and limited sample size warrant caution in translating the findings into definitive intervention priorities. We have revised the conclusion to temper the policy implications and present them as potential areas for consideration rather than firm recommendations. We also emphasize that the observed relationships should be interpreted as associations and that further population-based and longitudinal studies are needed to confirm the findings.

**Minor comments**

1. Please correct typographical errors, including “fruit ang vegetable intake.”

**Response:**

Corrected throughout the manuscript.

2. Please ensure consistency in terminology. The manuscript uses “poor physical activity,” “physical inactivity,” “smoking,” and “harmful smoking.” It would be clearer to use consistent labels throughout.

**Response:**

We have standardized terminology throughout the manuscript to improve clarity and consistency. Specifically, we replaced "poor physical activity" with "physical inactivity," "harmful smoking" with "current tobacco smoking," and consistently used "low fruit and vegetable intake" and "harmful alcohol use" throughout the text, tables, and supplementary materials. This terminology is aligned with WHO STEPS and public health reporting conventions.

3. The alcohol use definition would benefit from clarification. The manuscript defines excessive drinking as three or more drinks per day, approximately 60 g of alcohol. Please explain how this definition aligns with WHO STEPS or other established thresholds.

**Response:**

We have clarified the definition of harmful alcohol use in the Methods section. Specifically, we explain that the measure was derived from the WHO STEPwise Approach to NCD Risk Factor Surveillance (WHO STEPS), in which a standard drink contains approximately 20 g of pure alcohol. Consumption of three or more standard drinks per day (approximately 60 g of pure alcohol) was used to identify harmful alcohol use, consistent with established thresholds for heavy alcohol consumption and NCD risk.

4. Please clarify whether “low fruit intake” in the results and Table 1 refers to fruit only or combined fruit and vegetable intake, since other sections refer to low fruit and vegetable intake. Also please make sure the terminology is consistent (low or poor intake)

**Response:**

The variable was defined using combined fruit and vegetable consumption (<5 servings per day), not fruit consumption alone. To avoid ambiguity, we have revised the manuscript to consistently use the term "low fruit and vegetable intake" throughout the text, tables, and results sections. We have also replaced the term "poor fruit and vegetable intake" with "low fruit and vegetable intake" to ensure consistency in terminology.

We sincerely thank the Editor and Reviewers for their constructive comments, which have substantially improved the quality and clarity of the manuscript.
