# Supplementary material for: Factors associated with socioeconomic inequalities in the prevalence of chronic NCD risk factors in the DIMAMO surveillance site, South Africa
Source: PLOS Glob Public Health. 2026 Oct 26;6(10):e0007483. doi: 10.1371/journal.pgph.0007483 (PMC13704811; doi:10.1371/journal.pgph.0007483)
Supplement: Peer review history

# Author comment: Author response to Decision Letter 0

**DOI:** 10.1371/journal.pgph.0007483.r001

11 Dec 2025

Attachment Submitted filename: PLOS Response Letter.pdf

# Peer review: Decision Letter 0

**DOI:** 10.1371/journal.pgph.0007483.r002

**Authors:** Ali Aahil Noorali

19 May 2026

PGPH-D-25-03760

Determinants of socioeconomic inequalities in the prevalence of chronic NCDs risk factors in the DIMAMO surveillance site, South Africa

PLOS Global Public Health

Dear Dr. Ravhuhali,

Thank you for submitting your manuscript to PLOS Global Public Health. After careful consideration, we feel that it has merit but does not fully meet PLOS Global Public Health’s publication criteria as it currently stands. Therefore, we invite you to submit a revised version of the manuscript that addresses the points raised during the review process.

Please submit your revised manuscript by Jul 18 2026 11:59PM. If you will need more time than this to complete your revisions, please reply to this message or contact the journal office at globalpubhealth@plos.org. When you're ready to submit your revision, log on to https://www.editorialmanager.com/pgph/ and select the 'Submissions Needing Revision' folder to locate your manuscript file.

Please include the following items when submitting your revised manuscript:

- A letter that responds to each point raised by the editor and reviewer(s). You should upload this letter as a separate file labeled 'Response to Reviewers'.
- A marked-up copy of your manuscript that highlights changes made to the original version. You should upload this as a separate file labeled 'Revised Manuscript with Track Changes'.
- An unmarked version of your revised paper without tracked changes. You should upload this as a separate file labeled 'Manuscript'.

Guidelines for resubmitting your figure files are available below the reviewer comments at the end of this letter.

As the corresponding author, your ORCID iD is verified in the submission system and will appear in the published article. PLOS supports the use of ORCID, and we encourage all coauthors to register for an ORCID iD and use it as well. Please encourage your coauthors to verify their ORCID iD within the submission system before final acceptance, as unverified ORCID iDs will not appear in the published article. *Only*  the individual author can complete the verification step; PLOS staff *cannot*  verify ORCID iDs on behalf of authors.

We look forward to receiving your revised manuscript.

Kind regards,

Ali Aahil Noorali, MD, MPH, MBA

Guest Editor

PLOS Global Public Health

**Journal Requirements:**

1. In the online submission form, you indicated that “All data generated and analyzed from this investigation are available upon

reasonable request from the corresponding author.”.

All PLOS journals now require all data underlying the findings described in their manuscript to be freely available to other researchers, either

1. In a public repository,

2. Within the manuscript itself, or

3. Uploaded as supplementary information.

This policy applies to all data except where public deposition would breach compliance with the protocol approved by your research ethics board. If your data cannot be made publicly available for ethical or legal reasons (e.g., public availability would compromise patient privacy), please explain your reasons by return email and your exemption request will be escalated to the editor for approval. Your exemption request will be handled independently and will not hold up the peer review process, but will need to be resolved should your manuscript be accepted for publication. One of the Editorial team will then be in touch if there are any issues.

2. Please ensure that your Ethics Statement is available in its entirety at the beginning of your Methods section, under a subheading 'Ethics Statement'

If the reviewer comments include a recommendation to cite specific previously published works, please review and evaluate these publications to determine whether they are relevant and should be cited. There is no requirement to cite these works unless the editor has indicated otherwise.

**Additional Editor Comments (if provided):**

Sorry for the delay on your paper, we were awaiting a final report before communicating a decision to you. All of the reviewers have recommended some revisions and have provided detailed comments. Feel free to include them/not, as appropriate, based on your vision for the paper. These suggestions and comments should only be thought of as potential ways to improve your paper, but not as mandatory requirements/inclusions/exclusions. Good luck, and we look forward to reading the revised version.

Ali Aahil Noorali, MD, MPH, MBA

Johns Hopkins School of Medicine

[Note: HTML markup is below. Please do not edit.]

Reviewers' comments:

Reviewer's Responses to Questions

**Comments to the Author**

1. Does this manuscript meet PLOS Global Public Health’s publication criteria?

Reviewer #1: Yes

Reviewer #2: Partly

Reviewer #3: Partly

**********

2. Has the statistical analysis been performed appropriately and rigorously?

Reviewer #1: No

Reviewer #2: Yes

Reviewer #3: No

**********

3. Have the authors made all data underlying the findings in their manuscript fully available (please refer to the Data Availability Statement at the start of the manuscript PDF file)?

The PLOS Data policy

Reviewer #1: No

Reviewer #2: No

Reviewer #3: No

**********

4. Is the manuscript presented in an intelligible fashion and written in standard English?

Reviewer #1: Yes

Reviewer #2: Yes

Reviewer #3: No

**********

**Reviewer #1:**  This is an important report on the prevalence of NCDs and their distribution per chosen socioeconomic categories. The authors used cross-sectional data from an HDSS site in South Africa collected through the STEPS methodology. In general, the report is well written. However, there are issues with the analysis and presentation of results.

1. Analysis:

- This is an inequality analysis. Typically, in such analyses, we use an absolute measure of association (e.g., a proportion or prevalence difference). And if we use a relative measure of association, we use proportion ratios (or prevalence ratios). The odds ratio, as the authors chose here, is not the recommended measure of association for exploring inequality. Please reconsider this choice. I would suggest presenting unadjusted absolute differences and replacing the OR with proportion ratios (PR).

- Please do not use p-values to consider what should be adjusted for. Confounding is not about p-values, and you have only 6 covariates. Gender, age, education, and socioeconomic status should be part of the minimal set of covariates to keep.

2. Results:

- Table 1:

- present a row of totals

- Reconsider the age categories and add median and IQR

- Please revise the parentheses for the proportions in Alcohol consumption (eg: Q1 and Q2 columns)

- We do not need p-values on this table. But if you included, why did we miss the p-value for “Low fruit intake”? Please add below the table how these p-values were calculated.

- Table 3: For categories with zeros, just put a dash. Not a number. This is for the employment status for student.

- Table 7: For categories with zeros, just put a dash. Not a number. This is for the education status for tertiary. And marital status for divorced.

3. The same goes for the abstract. Be careful to speak of inequalities and use OR in the abstract.

**Reviewer #2:**  Overall Assessment

This manuscript presents a timely and relevant cross-sectional study examining the socioeconomic patterning of non-communicable disease (NCD) risk factors in a rural South African population. The research addresses an important evidence gap in a low-resource setting undergoing epidemiological transition. The study is well-conceived, uses standardized instruments, and applies appropriate statistical methods. However, several methodological and presentation issues must be addressed before the manuscript can be considered for publication in a high-impact journal.

Major concerns:

1. Data Collection Date Discrepancy

The manuscript text (Section 2.2) states data collection occurred from 21 August to 11 October 2024, but the author’s note to the editor indicates this was erroneously listed as 2025 in submission materials. This must be explicitly corrected in the manuscript (Abstract, Methods, and any relevant footnotes) to maintain credibility. A brief statement in the Methods or as a footnote should clarify and confirm that ethics approval covered the correct period.

2. Sample Size and Power Limitations

The sample size (n=398) is modest, especially when stratified into SES quintiles and multiple risk factors. This likely limits the power to detect significant associations, particularly for less prevalent outcomes (e.g., diabetes n=28). The authors should:

Discuss this limitation more explicitly in the Discussion.

Consider presenting confidence intervals consistently and noting where wide CIs indicate uncertainty (e.g., diabetes aOR: 1.5–18.1).

Avoid overinterpreting non-significant findings.

3. Measurement and Bias

Self-reported data for hypertension, diabetes, diet, and salt intake are subject to recall and social desirability bias. This should be highlighted as a key limitation.

Wealth index construction: The description is brief. Provide more detail on how the index was derived (PCA? simple sum?), which assets were included, and how quintiles were defined. This is crucial for replicability and interpretation.

Salt intake measurement: The method is not clearly described. How was high salt intake defined? If based on self-reported addition or consumption of processed foods, this should be explicitly stated and its limitations acknowledged.

4. Interpretation of Findings

The finding that higher SES is linked to higher diabetes risk is intriguing but should be contextualized within the epidemiological transition framework. The authors should more deeply discuss potential mechanisms (e.g., dietary shift, sedentary behavior) and contrast with patterns in high-income countries.

The association between secondary education and lower fruit/vegetable intake is counterintuitive and merits more nuanced discussion (e.g., education without economic empowerment, food environment constraints).

**Reviewer #3:**  Thank you for the opportunity to review this manuscript. The study addresses an important public health question. Overall, the manuscript has the potential to contribute to the literature on NCD risk factor distribution in rural LMIC settings. However, several methodological, analytical, and reporting issues should be addressed to improve the clarity and strength of the manuscript before publication.

Major comments

1. Clarify study design and representativeness

The manuscript describes the study as being conducted within the DIMAMO HDSS, but the methods indicate that participants were recruited from selected primary healthcare facilities, with the sampling frame limited to community members presenting to the clinics on data collection days. This should be clearly described as a facility-based sample within the DIMAMO HDSS area, unless the authors can justify that it represents the broader HDSS population. Because PHC attendees may differ from the general population in health status, healthcare-seeking behavior, diagnosis history, and socioeconomic profile, the implications for selection bias and generalizability should be discussed.

2. Provide more detail on the wealth index

SES is central to the study objective and conclusion, but the construction of the wealth index is not sufficiently clear. Please describe the exact approach used to derive the wealth score (for instance principal component analysis, equal weighting, summed scoring), how items were selected, how missing values were handled, and why the reported quintile cutoffs were chosen. Providing a methodological justification and relevant reference would strengthen reproducibility and interpretation.

3. Address small event counts and statistical precision

Some outcomes had few events, like self-reported diabetes with only 28 cases. Given the number of covariates in the adjusted model and the wide confidence intervals, this should be added to the limitations, and the diabetes-related findings should be interpreted more cautiously.

4. Clarify covariate selection and model-building strategy

The methods section states that variables with p < 0.20 in univariate models were considered for multivariable models, while SES was retained regardless of significance. Please clarify whether this was based on the “purposeful selection approach” and provide an appropriate reference for the screening threshold. It is also not clear whether the same covariate set was used across all outcomes or whether each outcome had a separate model-building process. Please clarify how final covariates were selected for each adjusted model and whether selection was based only on statistical criteria or also informed by conceptual/confounding considerations.

5. Interpret cross-sectional associations more cautiously

Given the cross-sectional design, the manuscript cannot establish temporality or causality. The authors should avoid causal language such as “drivers,” or “determinants” unless carefully qualified. I suggest reframing the title, objective, discussion, and conclusion to use terms such as “factors associated with” or “correlates of” socioeconomic inequalities in NCD risk factors. Findings should be interpreted as associations only.

6. Self-reported hypertension and diabetes may introduce detection bias

Hypertension and diabetes were self-reported. This is an important limitation in an SES analysis, because higher-SES participants may have better access to screening and may therefore be more likely to know and report a diagnosis. The observed positive association between higher SES and diabetes may partly reflect differential diagnosis or awareness rather than true disease prevalence. This should be discussed more explicitly.

7. Discussion could be strengthened conceptually

The discussion appropriately compares findings with prior literature, but it could be more analytical regarding the mixed SES patterns observed. For example, higher SES was associated with higher odds of diabetes, harmful alcohol use, and high salt intake, but lower odds of smoking and physical inactivity. While a lower SES was associated with smoking and physical inactivity. Therefore, the SES gradient is not uniform across all NCD risk factors. A brief explanation of why higher SES may be associated with some risks but appear protective for others would strengthen the interpretation and help contextualize the findings in rural South Africa.

8. Avoid overgeneralized policy recommendations

The conclusion recommends targeted interventions by SES group. While this is reasonable, the recommendations should be tempered given the facility-based sample, cross-sectional design, self-reported measures, and limited sample size. The authors should present these as potential implications rather than definitive intervention priorities.

Minor comments

1. Please correct typographical errors, including “fruit ang vegetable intake.”

2. Please ensure consistency in terminology. The manuscript uses “poor physical activity,” “physical inactivity,” “smoking,” and “harmful smoking.” It would be clearer to use consistent labels throughout.

3. The alcohol use definition would benefit from clarification. The manuscript defines excessive drinking as three or more drinks per day, approximately 60 g of alcohol. Please explain how this definition aligns with WHO STEPS or other established thresholds.

4. Please clarify whether “low fruit intake” in the results and Table 1 refers to fruit only or combined fruit and vegetable intake, since other sections refer to low fruit and vegetable intake. Also please make sure the terminology is consistent (low or poor intake)

**********

what does this mean?). If published, this will include your full peer review and any attached files.

**Do you want your identity to be public for this peer review?**  If you choose “no”, your identity will remain anonymous but your review may still be made public.

For information about this choice, including consent withdrawal, please see our Privacy Policy

Reviewer #1: No

Reviewer #2: **Yes:**  Palash Chandra Banik

Reviewer #3: No

**********

[NOTE: If reviewer comments were submitted as an attachment file, they will be attached to this email and accessible via the submission site. Please log into your account, locate the manuscript record, and check for the action link "View Attachments". If this link does not appear, there are no attachment files.]

**Figure Resubmissions:**

Attachment Submitted filename: Peer review PLOS (GPH).docx

# Author comment: Author response to Decision Letter 1

**DOI:** 10.1371/journal.pgph.0007483.r003

27 Jul 2026

Attachment Submitted filename: Response to Reviewers PLOS (GPH)_26Jul2026.docx

# Peer review: Decision Letter 1

**DOI:** 10.1371/journal.pgph.0007483.r004

**Authors:** Ali Aahil Noorali

30 Sep 2026

Factors associated with socioeconomic inequalities in the prevalence of chronic NCD risk factors in the DIMAMO surveillance site, South Africa

PGPH-D-25-03760R1

Dear Mr Ravhuhali,

We are pleased to inform you that your manuscript 'Factors associated with socioeconomic inequalities in the prevalence of chronic NCD risk factors in the DIMAMO surveillance site, South Africa' has been provisionally accepted for publication in PLOS Global Public Health.

Before your manuscript can be formally accepted you will need to complete some formatting changes, which you will receive in a follow up email. A member of our team will be in touch with a set of requests.

Please note that your manuscript will not be scheduled for publication until you have made the required changes, so a swift response is appreciated.

IMPORTANT: The editorial review process is now complete. PLOS will only permit corrections to spelling, formatting or significant scientific errors from this point onwards. Requests for major changes, or any which affect the scientific understanding of your work, will cause delays to the publication date of your manuscript.

If your institution or institutions have a press office, please notify them about your upcoming paper to help maximize its impact. If they'll be preparing press materials, please inform our press team as soon as possible -- no later than 48 hours after receiving the formal acceptance. Your manuscript will remain under strict press embargo until 2 pm Eastern Time on the date of publication. For more information, please contact globalpubhealth@plos.org.

Thank you again for supporting Open Access publishing; we are looking forward to publishing your work in PLOS Global Public Health.

Best regards,

Ali Aahil Noorali, MD, MPH, MBA

Guest Editor

PLOS Global Public Health

***********************************************************

Reviewer Comments (if any, and for reference):

Reviewer's Responses to Questions

**Comments to the Author**

Reviewer #1: All comments have been addressed

Reviewer #2: All comments have been addressed

Reviewer #3: All comments have been addressed

**********

publication criteria?

Reviewer #1: Yes

Reviewer #2: Yes

Reviewer #3: Yes

**********

3. Has the statistical analysis been performed appropriately and rigorously?

Reviewer #1: Yes

Reviewer #2: Yes

Reviewer #3: Yes

**********

4. Have the authors made all data underlying the findings in their manuscript fully available (please refer to the Data Availability Statement at the start of the manuscript PDF file)?

The PLOS Data policy

Reviewer #1: Yes

Reviewer #2: Yes

Reviewer #3: Yes

**********

5. Is the manuscript presented in an intelligible fashion and written in standard English?

Reviewer #1: Yes

Reviewer #2: Yes

Reviewer #3: Yes

**********

**Reviewer #1** : The authors addressed all my previous comments.

Two small issues:

1. In the table 1 - please report median and IQR for age for each of the SES quintile.

2. In the discussion, second paragraph it seems to be confusion between "population demographic structure" and "health care provision/utilization". So the kind of population seen at the health facility cannot be completely due to one of those components.

**Reviewer #2:**  I am happy with the revised manuscript.

**Reviewer #3** : Thank you for addressing my comments from previous round. The revised manuscript is clearer and I have no further comments.

**********

what does this mean?). If published, this will include your full peer review and any attached files.

**Do you want your identity to be public for this peer review?**  If you choose “no”, your identity will remain anonymous but your review may still be made public.

For information about this choice, including consent withdrawal, please see our Privacy Policy

Reviewer #1: No

Reviewer #2: **Yes:**  Palash Chandra Banik

Reviewer #3: No

**********
